# Efficacy of hydroxyapatite-based skull base reconstruction for intraoperative high-flow cerebrospinal fluid leakage performed by less-experienced surgeons

**DOI:** 10.1038/s41598-023-42097-y

**Published:** 2023-09-09

**Authors:** Inseo Hong, Kyung Hwan Kim, Youngbeom Seo, Yoon-Hee Choo, Han-Joo Lee, Seon-Hwan Kim

**Affiliations:** 1 Department of Neurosurgery, Chungnam National University Hospital, Chungnam National University School of Medicine, 282 Munhwa-ro, Jung-gu, Daejeon, 35015 South Korea; 2 Department of Neurosurgery, Yeungnam University Hospital, Yeungnam University College of Medicine, Daegu, South Korea; 3 Department of Neurosurgery, Seoul St. Mary’s Hospital, The Catholic University of Korea College of Medicine, Seoul, South Korea

**Keywords:** Neurology, Oncology

## Abstract

Cerebrospinal fluid (CSF) leakage after endoscopic skull base surgery remains a challenge despite multilayer reconstruction including nasoseptal flap (NSF) has become a standard technique. Injectable hydroxyapatite (HXA) has shown promising results to prevent CSF leakage. This study aimed to validate the efficacy of HXA-based skull base reconstruction performed by less-experienced neurosurgeons who had short-term clinical experiences as independent surgeons. Between March 2018 and November 2022, 41 patients who experienced intraoperative high-flow CSF leakage following endoscopic endonasal surgery at two independent tertiary institutions were enrolled. Skull base reconstruction was performed using conventional multilayer techniques combined with or without HXA. The primary outcome was postoperative CSF leakage. The surgical steps and nuances were described in detail. The most common pathology was craniopharyngioma. Injectable HXA was used in 22 patients (HXA group) and conventional techniques were performed in 19 patients (control group). The HXA group achieved a significantly lower incidence of postoperative CSF leakage than the control group (0% vs. 26.3%, p = 0.016). No HXA-related complications were observed. The use of injectable HXA in skull base reconstruction was highly effective and safe. This technique and its favorable results might be readily reproduced by less-experienced neurosurgeons.

## Introduction

The transsphenoidal approach (TSA) has been used for the removal of tumors located within the sellar region^1^. In the late 1980s, an extended TSA was adopted and has shown the potential to gain access to the suprasellar and/or parasellar area^2^. More recently, a pure endoscopic technique has expanded the applications of this surgical approach^3^. However, this approach has several limitations, including a narrow surgical view and/or postoperative cerebrospinal fluid (CSF) leakage^2,4^. Despite recent technical advancements in endoscopic skull base surgery, CSF leakage remains one of the most challenging concerns, especially for less-experienced surgeons. The overall CSF leakage rate in the literature varied widely according to tumor characteristics, location, surgical techniques, and surgeons’ expertise^5–7^.

Multilayered skull base reconstruction is now a standard technique for repairing skull base defects. The goal of skull base reconstruction for CSF leakage is to achieve watertight closure until the scar formation seals the defect. To date, a variety of reconstruction techniques combined with various materials, including fat, fascia, or vascularized mucosal flaps, has been devised^5,8–11^. Among these materials, injectable hydroxyapatite (HXA) has shown promising results for the surgical repair of CSF leakage^12–14^. HXA is a suitable reconstruction material for repairing bony defects owing to its properties, such as easy incorporation into the bone defect due to its injectable consistency, volumetric stability over time, biocompatibility, mechanical strength, and minimal inflammatory response^12,15^. Recently, several studies have shown favorable clinical outcomes using injectable HXA to prevent CSF leakage^14,16,17^. However, to date, little has been reported on the possibility of applying the HXA-based reconstruction technique by surgeons in the middle of learning curve to minimize the CSF leakage rate. This study aimed to assess the efficacy of HXA-based skull base reconstruction performed by less-experienced neurosurgeons at the two institutions.

## Materials and methods

### Study design and patient characteristics

This multicenter retrospective study was conducted by two neurosurgeons (Y.S. and K.H.K.) at each independent tertiary institution. Both neurosurgeons performed all endoscopic procedures, from the nasal phase to the reconstructive phase by themselves. At the time of data collection, they had similar clinical experiences of 4–5 years as independent surgeons without supervisors and were on a steep learning curve. The study inclusion criteria were patients who underwent multilayer skull base reconstruction, including a nasoseptal flap (NSF) for high-flow grade 3 CSF leakage following endoscopic endonasal surgery^18^. Patients with grade 0 to 2 intraoperative CSF leakage who underwent reconstruction without NSF were excluded.

Patient data, including demographic, clinical, and surgical information, were consecutively collected between March 2018 and November 2022. Since 2021, the recent period of the study, most of the patients underwent skull base reconstruction using an injectable HXA (Hydroset®; Stryker, Kalamazoo, MI, USA) with NSF (HXA group). Dynamic magnetic resonance (MR) images with gadolinium contrast on a 3 T scanner and computed tomography (CT) scans and angiography with 1 mm slices were obtained from all patients preoperatively to verify the details of the nasal cavity, tumor, and vascular anatomy. Preoperative basal hormone tests and ophthalmological examinations were routinely performed in all patients.

The primary outcome was postoperative CSF leakage requiring neurosurgical interventions based on the findings of the endoscopic nasal examination. In addition, the extent of resection, total operative time, surgical complications, nasal complications, and postoperative central nervous system infections were investigated. This study was approved by the local institutional review board of Chungnam National University Hospital (IRB no. CNUH-2023-03-067). The study is in accordance with the Declaration of Helsinki and informed consent was waived.

### Surgical procedures

Endoscopic endonasal surgery was performed as usual, and detailed procedures have been described in the literature^16,19^. All surgeries were performed using a direct 4-mm 0° endoscope and 30° or 45° angled endoscope (Karl Storz Co., Tuttlingen, Germany) under the guidance of a neuronavigation system (Medtronic, Minneapolis, USA). In the nasal phase, a vascularized NSF was raised at the beginning of the surgery and prepared for skull base reconstruction. A modified transseptal TSA was used to allow binostril surgery and to reduce sinonasal morbidity by preserving the nasal mucosa^20^. The anterior septal mucosa, which was dissected in a submucoperichondrial and submucoperiosteal fashion toward the rostrum sphenoidale on the contralateral side of the NSF harvesting side, was tagged to the nostril (left side in most cases). When unexpected high-flow CSF leakage developed during surgery, the NSF was raised from the rescue flap after tumor removal.

**Figure 1:**
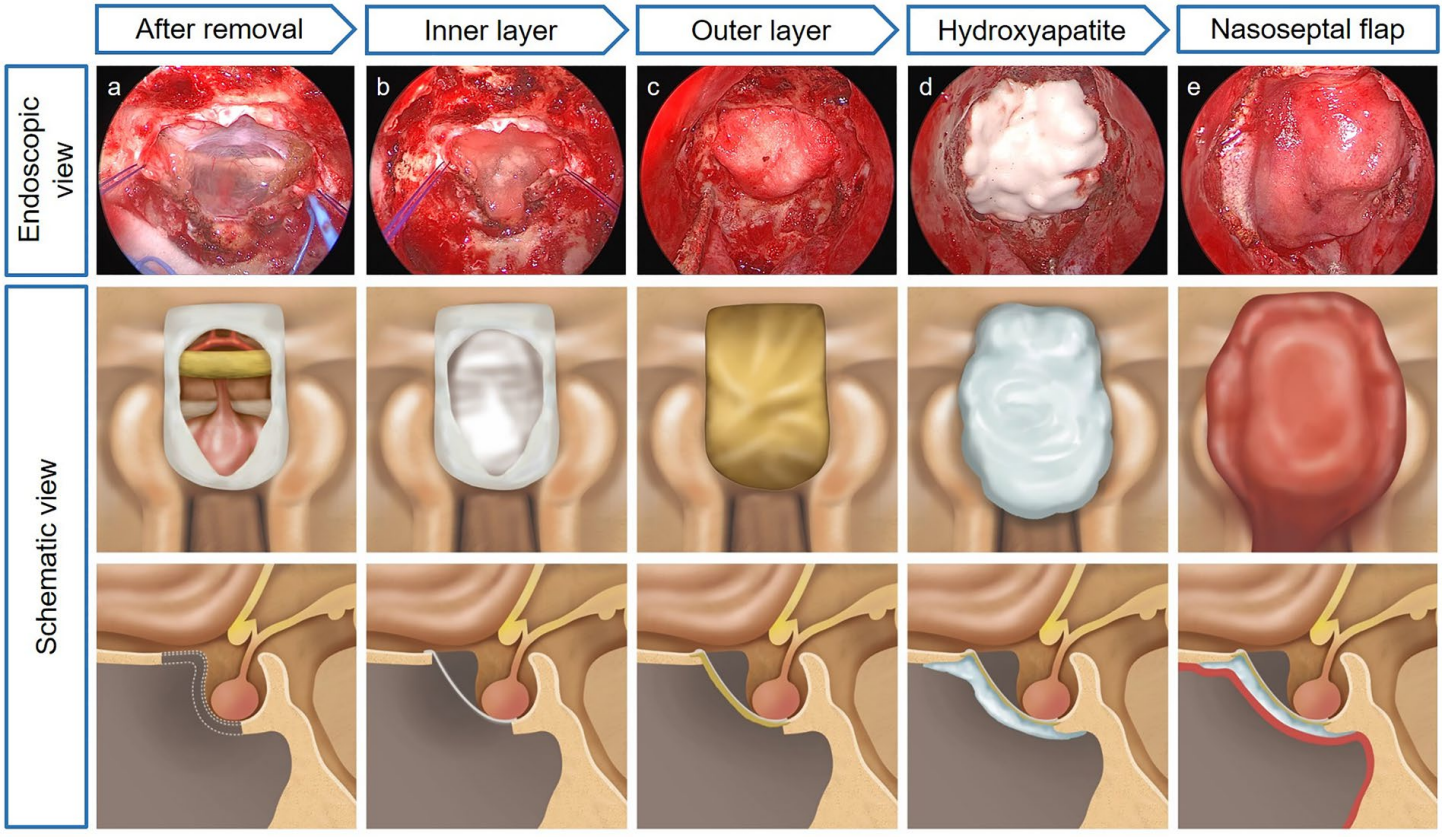
Endoscopic and schematic images of hydroxyapatite-based skull base reconstruction; There are endoscopic images for each step in the first column (**a**–**e**), and corresponding illustrations in the second and third columns; (**a**) After tumor removal, meticulous hemostasis is confirmed before the reconstruction; (**b**) the collagen matrix inlay is placed on the intracranial side of the dura mater; (**c**) A tailored acellular dermal graft is inserted between dura and bone defect or covered bone defect as outlay material which is reinforced by fibrin sealant patches; (**d**) Injectable hydroxyapatite is applied to seal the bony defect; (**e**) After ensuring there is no cerebrospinal fluid leakage, a vascularized nasoseptal flap is placed to cover all the corners of the construct.

After tumor resection, meticulous hemostasis, and complete skeletonization around the skull defect, multilayer skull base reconstruction was performed (Fig. 1). First, the inlay material was placed on the intracranial side of the dura mater. A collagen matrix (Duragen®; Integra LifeScience, New Jersey, USA) was the most frequently used material. Autologous fat grafts were used in the early study period but were never used in the late period. The incised dura was loosely re-approximated and sutured if possible. It is not for the watertight dural closure, but for the stabilization of intracranial constructs. The outlay material was then applied to the dura and placed between the margin of the bony defect and the dura mater. In most cases, a 1 mm-thin acellular dermal graft (MegaDerm®; L&C BIO, Gyeonggi-do, Korea) was used as an outlay material. Generally, the tucking of the outlay material in the skull defect was not perfect. Therefore, a fibrin sealant patch (Tachosil®; Takeda Pharma, Linz, Austria) was attached to the margin of the outlay graft and the skull base defect. Securing a sufficiently denuded bony margin to contact the HXA and NSF was necessary after applying the fibrin sealant patches.

**Figure 2:**
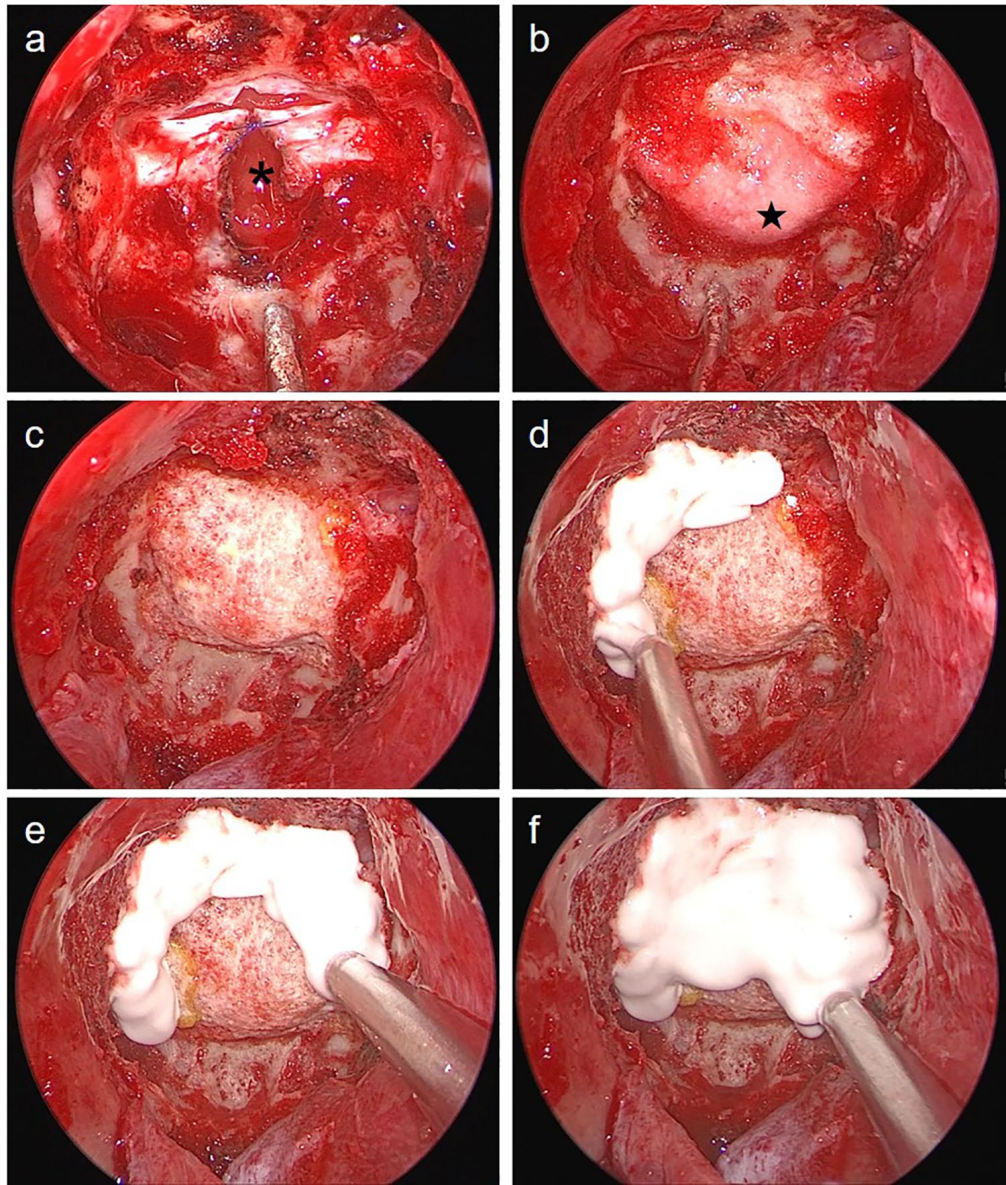
Surgical nuances for preparing and injecting hydroxyapatite; (**a**) the incised dura is re-approximated with sutures for stabilization of the collagen matrix inlay (asterisk) during reconstruction; (**b**) The margins of the outlay graft (star) and bone defects are sealed with fibrin sealant patches since the tucking of the outlay graft is generally imperfect; (**c**) Drying the bony surface before using an injectable hydroxyapatite is essential; (**d**–**f**) To cover the entire construct without defects, injection is started from the margins to the center.

Drying the bony surface before using an injectable HXA is mandatory. Therefore, the sphenoid sinus and nasal cavity should be packed with 1:10,000 epinephrine-soaked gauzes and cottonoid patties for a sufficient time. Injection of HXA started from the margins of the skull base defect and construct, and semi-solid HXA had to be in contact with the bony surface. After covering all the margins, it was injected over the central side. This order of injection was helpful for completely covering the target without HXA defects (Fig. 2). During HXA injection and hardening, care should be taken to prevent contamination of blood from the nasal cavity. Close surveillance for 10–15 min during hardening was necessary to prevent graft failure. After the completion of the process, the surface of the HXA construct was gently touched to confirm sufficient hardness. At this time, positive ventilation was performed at least twice using the Valsalva maneuver to evaluate the firmness of the reconstruction. Then, a vascularized NSF covered all the corners of the HXA constructs and was attached to the bony surface. To avoid the unexpected migration of the NSF, pieces of oxidized cellulose (Surgicel®; Johnson and Johnson, New Brunswick, NJ, USA) were spread in a thin layer along the margin of NSF following the application of fibrin sealant (Tisseel®; Baxter, Illinois, USA) or hydrogel sealant (DuraSeal®; Confluent Surgical, Inc., Waltham, MA, USA). The Valsalva maneuver was repeated to confirm a watertight seal. Using nasal dressing material (Merocel®; Medtronic, Minneapolis, USA), the sphenoid sinus was gently filled without pressure. In HXA-based reconstruction, it was not necessary to compress the NSF with dressing materials, absorbable gelatin sponges, fat, or balloon catheters. The surgical steps of the reconstruction procedure are demonstrated in Supplementary Video 1.

In the cases of conventional multilayer reconstruction (control group), autologous fat and/or fascia were commonly used instead of synthetic materials. The most frequently used method was the bilayer button technique using a sutured fascial construct, followed by the gasket seal technique with an autologous septal bone graft^11,21^. The reconstructions were then covered by an NSF and secured with a fibrin sealant. In contrast to HXA-based reconstruction, tight compression against the NSF by various materials was essential. In addition, postoperative LD was placed when undergoing posterior fossa surgery and when the third ventricle was opened during surgery. Prophylactic antibiotics, including 3rd generation cephalosporin and quinolone, were administered for a duration of 3 to 5 days.

### Postoperative care and follow-up

Postoperative CT was performed immediately after surgery to determine the state of reconstruction and surgical complications. MR images, including sagittal and coronal sections of T1 contrast enhancement and T2-weighted turbo spin echo sequences, were acquired within 24–48 h after surgery to evaluate the viability of NSF and CSF leakage. Postoperatively, head elevation over 30 degrees was rigorously maintained and ambulation was encouraged to lower intracranial pressure as well as to prevent deep vein thrombosis and lung complications. The nasal cavity was examined endoscopically 2 and 5 days after surgery to assess NSF viability and CSF leakage. The packing materials were removed at five days after surgery. Patients without fever or uncontrolled electrolyte imbalance were discharged 6–7 days after surgery. Outpatient nasal cavity examinations were performed at 1 to 2–week intervals thereafter until the mucosa completely healed without complications. If CSF leakage was evident, neurosurgical interventions including CSF diversion and re-operation was immediately performed.

### Statistical analysis

Categorical variables are presented as number of patients with percentage. The Fisher’s exact test was used in comparing categorical variables. Parametric and nonparametric continuous variables presented as the mean values with standard deviation and the median values with range, respectively. The independent samples *t* test and the Mann–Whitney *U*-test were used in comparing continuous variables. Logistic regression analysis was used for univariate and multivariate analyses for CSF leakage. All statistical analyses were performed using R software (version 4.1.2; R foundation for statistical computing, Vienna, Austria), and statistical significance was set at p < 0.05.

## Results

### Baseline characteristics

This study investigated 41 patients who underwent multilayer skull base reconstruction with or without HXA for grade 3 CSF leakage following endoscopic endonasal surgery. The institutions enrolled 22 (53.7%) and 19 (46.3%) patients, respectively. The median age was 55 years (range, 8–80 years), and 15 patients (36.6%) were female. The most common pathology was craniopharyngioma (n = 19, 46.3%), followed by pituitary adenoma (n = 13, 31.7%), meningioma (n = 4, 9.8%), chordoma (n = 2, 4.9%), and others (n = 3, 7.3%; pituitary stalk hypophysitis, ectopic pituitary gland, and malignant melanoma). Among the 13 pituitary adenomas, seven were giant macroadenomas with a suprasellar extension to the third ventricle, and six were residual or recurrent lesions requiring re-operation. Prior transnasal surgery was performed in six patients (four microscopic and two endoscopic TSAs for pituitary adenoma). Regarding tumor location, 34 lesions (82.9%) were in the suprasellar region, and five (12.2%) were in the anterior skull base, and two (4.9%) were located in the clivus. In 36 patients (87.8%), gross or near-total resection was achieved and five patients (12.2%) had sub-total resection. The median follow-up time was 23.8 months (range, 2.0–57.4 months).

### Group comparison and reconstruction outcomes

According to the HXA use in multilayer skull base reconstruction, there were 22 patients (53.7%) in the HXA group and 19 patients (46.3%) in the control group. Both groups were balanced in age, sex, BMI, maximal diameter, and tumor location (Table 1). Autologous tissue grafts were not used in the HXA group, whereas seven patients (36.8%) in the control group underwent skull base reconstruction using abdominal fat and/or autologous fascia. Postoperative LD was used once (6.3%) in the HXA group and nine patients (47.4%) in the control group underwent prophylactic LD after surgery (p = 0.002).

**Table 1:** Patient characteristics and group comparison*.

Variables	HXA (n = 22)	Control (n = 19)	P-value
Age (years)	57 (16–73)	51 (8–80)	0.205
Female	7 (36.8)	8 (36.4)	1.000
Body mass index (kg/m^2^)	25.3 (4.1)	25.1 (4.2)	0.846
Maximal diameter (mm)	26.6 (10.7)	28.4 (6.4)	0.543
Tumor location			0.051
Suprasellar	16 (72.7)	18 (94.7)	
Anterior skull base	5 (22.7)	0 (0)	
Clivus	1 (4.5)	1 (5.3)	
Gross-total resection	20 (90.9)	16 (84.2)	0.649
Operation time (minute)	292 (200–480)	240 (175–505)	0.169
Autologous tissue grafts	0 (0)	7 (36.8)	0.002
Postoperative LD	1 (4.5)	9 (47.4)	0.002
Postoperative bleeding	1 (4.5%)	2 (10.5%)	0.588
Outcomes			
CSF leakage	0 (0)	5 (26.3)	0.016
Meningitis	0 (0)	3 (15.8)	0.091

*HXA* hydroxyapatite, *LD* lumbar drainage, *CSF* cerebrospinal fluid.

*Categorical variables are presented as number of patients with percentage; parametric and nonparametric continuous variables presented as the mean values with standard deviation and the median values with range, respectively.

**Figure 3:**
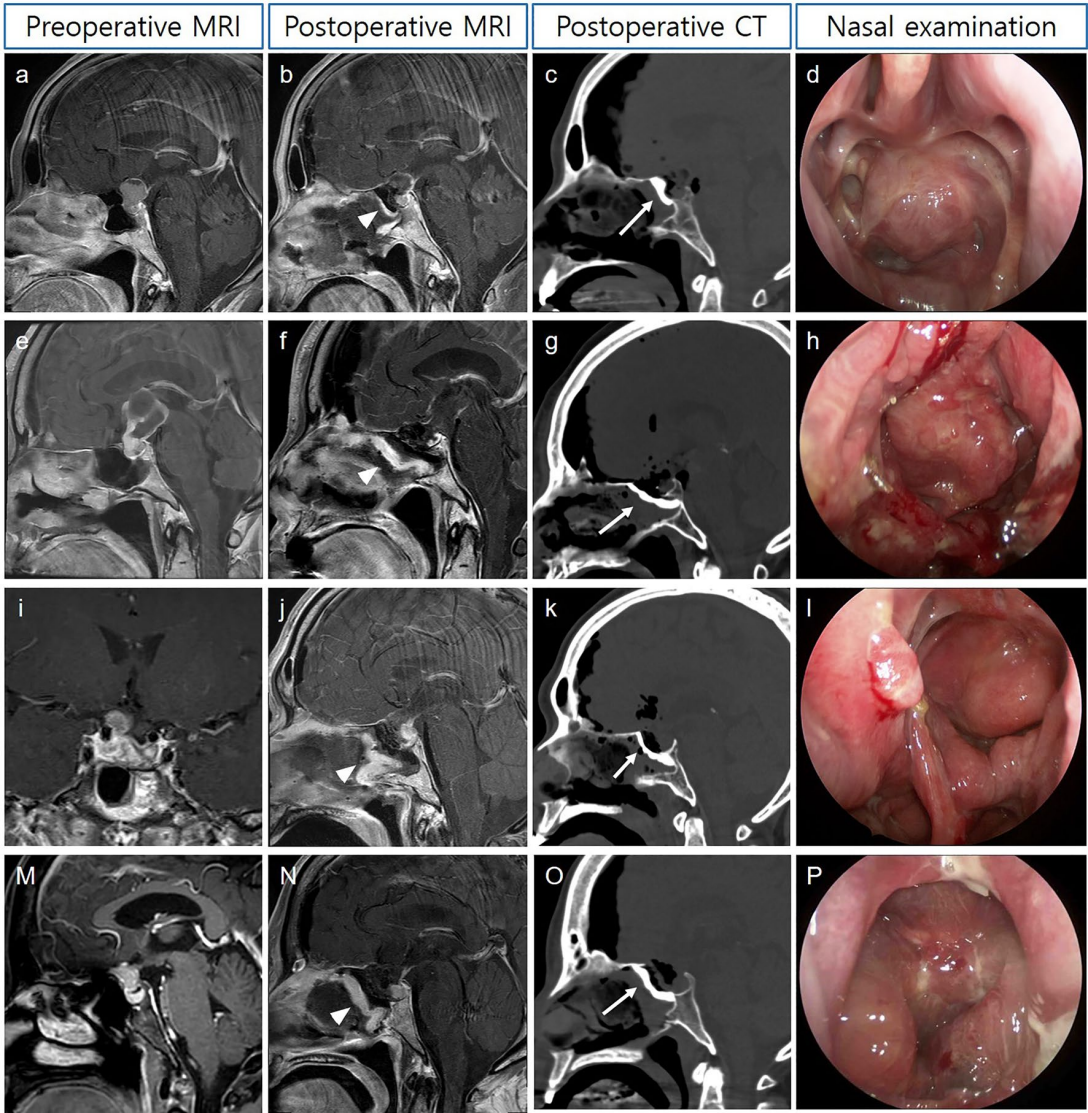
Representative cases of HXA-based skull base reconstruction; nasoseptal flaps (white arrow head) and hydroxyapatite layers (white arrow) are indicated (**a**) A 61-year-old female with craniopharyngioma presents with headache; (**b**) the tumor is completely removed via endoscopic trans-planum and trans-tubercular approach; note that a nasoseptal flap is well-enhanced (**c**) bony defect is reconstructed by the hydroxyapatite; (**d**) mucosalization is completed without complications; (**e**–**h**) A 62-year-old female with large craniopharyngioma underwent extended endoscopic surgery followed by hydroxyapatite-based skull base reconstruction; (**i**–**l**) A 52-year-old male presents a recurrent pituitary adenoma; the suprasellar portion of the tumor compressing the right optic nerve is completely removed by extended endoscopic surgery; (**m**–**p**) A case of 53-year-old female with tuberculum sellae meningioma treated with endoscopic endonasal surgery followed by hydroxyapatite-based skull base reconstruction.

**Figure 4:**
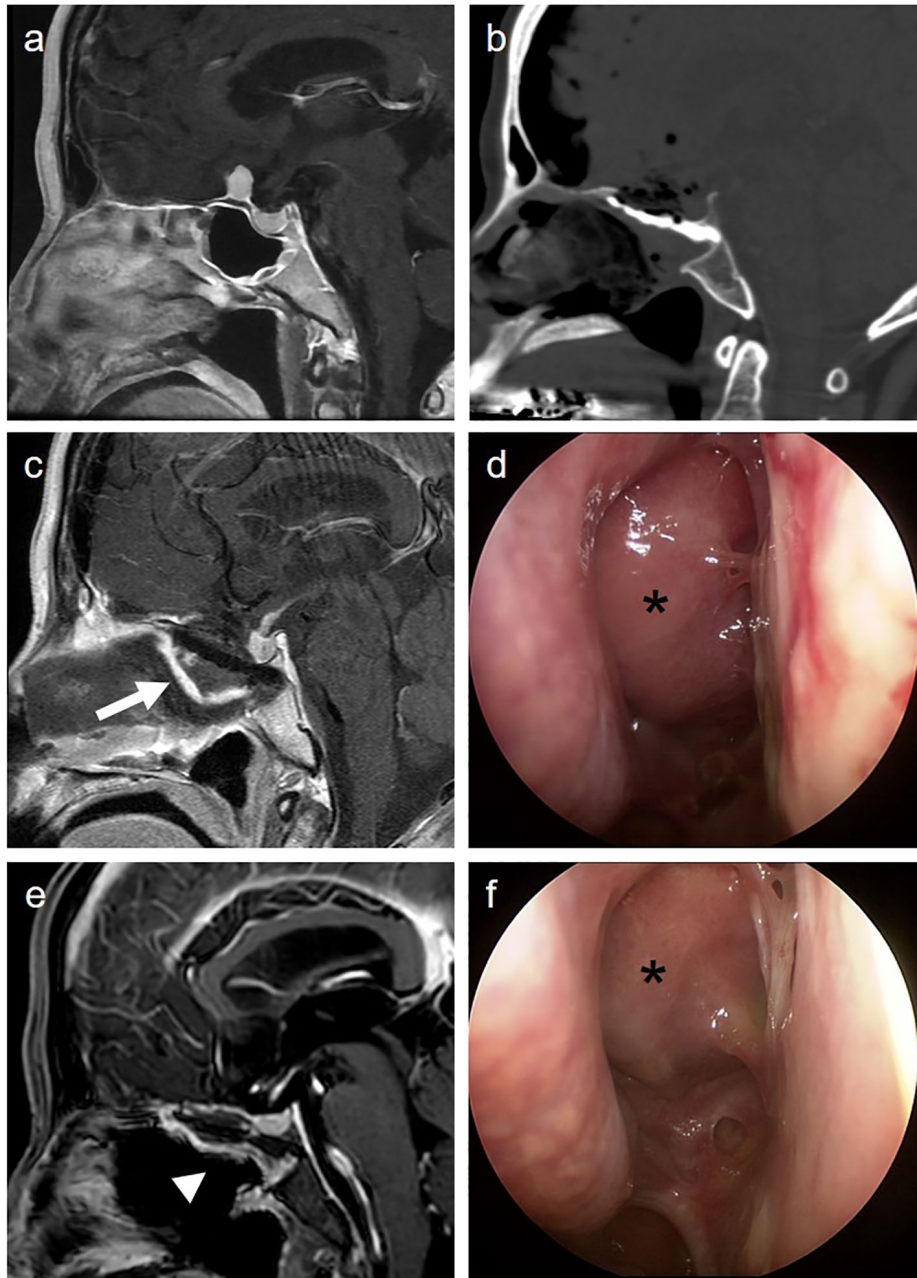
Spontaneous healing of improperly placed nasoseptal flap without cerebrospinal fluid leakage; (**a**) A 60-year-old female with a small planum sellae meningioma treated with endoscopic extended transsphenoidal approach; (**b**) The skull base defect is reconstructed using injectable hydroxyapatite; (**c**) Immediate postoperative image reveals a vascularized nasoseptal flap floating from the hydroxyapatite construct (white arrow); (**d**) Nasal endoscopic examination shows protrusion of the nasoseptal flap without cerebrospinal fluid leakage, pulsation, or flap necrosis (asterisks); (**e**) Four months after surgery, the nasoseptal flap is attached to the skull base (arrow head); (**f**) The mucosalization process is completed without complications.

Regarding the surgical outcomes, the extent of resection, operation time and hematoma in resection cavity were similar in the both groups (Table 1). However, postoperative CSF leakage rates were significantly improved in the HXA group. None of the patient (0%) the HXA group had CSF leakage (p = 0.016). In addition, no bacterial or aseptic meningitis was observed in the HXA group. All patients in the HXA group, except for one patient with a postoperative hematoma, were discharged a week after surgery. During routine outpatient nasal examinations, there were no unexpected events associated with mucosal healing (Fig. 3). There was one case in which the NSF floated from the HXA construct, but mucosalization progressed well over time without CSF leakage (Fig. 4). However, CSF leakage was evident in five patients (26.3%) in the control group, and three patients (15.8%) suffered from CSF leakage-related meningitis. Among the 9 patients of the control group, who underwent prophylactic LD, 3 (33.3%) experienced postoperative CSF leakage immediately after the removal of LD. On the other hand, 2 out of 10 patients (20.0%) without prophylactic LD in the control group showed CSF leakage one or two days after surgery. In all five patients with CSF leakage, the NSF was viable but was detached or displaced from the bony defect. Three patients underwent LD for 3 to 5 days after confirmation of CSF leakage, and two of them resolved. Two patients and one patient who was not treated with LD underwent reoperation. In multivariate analyses, HXA was the only positive factor that prevented CSF leakage (p = 0.009; odd ratio 0.771, 95% confidence interval = 0.636–0.928).

## Discussion

This retrospective multicenter study revealed the efficacy and safety of HXA-based skull base reconstruction. Neurosurgeons with limited experience achieved promising results with this new approach to prevent CSF leakage. According to this study, CSF leakage may no longer be an obstacle to the learning curve of endoscopic skull base surgery.

In this study, the HXA-based multilayer reconstruction technique effectively prevented CSF leakage without the incorporation of autologous tissue grafts or postoperative LD. Despite the remarkable progress over the past decades, the overall CSF leakage rate was 11.5% in the systematic review^22^. A recent meta-analysis showed that the overall rate of CSF leakage did not change over time^13^. Several authors have emphasized the learning curve of the surgeon and the importance of adherence to the basic techniques in skull base reconstruction^6,7,21,23,24^. In most centers, there is a decrease in postoperative CSF leakage as well as an increase in the rate of the extent of resection after the first 50 to 200 cases, after which the results tend to plateau^5–7,21,24–26^. Moreover, initial results from institutions currently at the expert level showed CSF leakage rates in the range of 23–60%^7,21,24,27^. However, it should be noted that these early results encompass cases performed prior to the establishment of reconstruction techniques. Of course, it takes time to master the skills, but the complications of CSF leakage pose serious risks to the patient^28^. Although both neurosurgeons in the study had fellowship in their brain tumor subspecialty and had performed nearly 100 endoscopic TSA without supervisors, the early surgical result of the extended endoscopic surgery was miserable. Even for surgeons less experienced in endoscopic skull base surgery, it is important to find a safe and reliable method to reduce the learning curves.

Since the introduction of injectable HXA, it can be applied in TSA because of the deep-located cranial defect within a narrow surgical corridor^12^. Kitano et al. used the multilayering method of injectable HXA to repair bone defects with successful results in 98% of patients^12^. Recently, several studies have reproduced successful outcomes, including the present study, using HXA-based reconstruction^14,16,17^. In this study, successful reconstruction could be predicted immediately after creating a rigid HXA layer, which was evaluated using the Valsalva maneuver. In addition to providing immediate mechanical strength for the reconstruction, HXA promotes bone ingrowth and provides biocompatibility, as the artificial HXA eventually converts to the major component of human skeletal bone^29^. Nevertheless, the potential risks of acute and chronic inflammation reaction associated with foreign materials are inherent. Several studies have reported favorable outcomes in terms of HXA-related complications^12,14,17,30^. However, Kim et al. reported that 13.5% of patients who underwent the HXA on-lay reconstruction technique had postoperative meningitis without definite microorganisms. They used a fibrin sealant patch alone on the dural defect as a temporary barrier to the CSF and then covered the bone defect and dura with an HXA layer. It is thought that multilayered isolation of the intradural space minimized contact between the HXA and CSF and played a role in reducing potential intracranial inflammatory reactions. Moreover, naked HXA exposure to the side of the nasal cavity accompanied by failure of mucosalization on the nasal side of the HXA is also a potential risk factor for intracranial inflammation. Jung et al. reported delayed mucosal coverage of a bare HXA implant with an average time of 14 weeks^30^. Complete NSF coverage over the HXA layer is essential to promote mucosalization of reconstructions and to reduce contact between the nasal cavity and HXA.

Since the introduction of vascularized NSF, the most important milestone in the evolution of skull base reconstruction, CSF leakage rates were apparently reduced using NSF^31,32^. However, multi-layer reconstruction with NSF does not completely block CSF leakage. Additionally, mechanical supports over the NSF by the proper buttresses, including fat graft, packing materials, or balloon catheter, were required to secure the time for NSF adhering to the defect site^5,18^. Moreover, CSF diversion through LD was frequently utilized despite its disadvantages, including patient immobilization, risk of infection, and CSF overdrainage^33^. Although a recent randomized study found that placement of postoperative LD was effective, the authors recommended LD only for patients with large anterior or posterior fossa intradural defects and not for small defects in the suprasellar region^34^. Conger et al. described that “LD may provide a ‘false sense of security’ to the surgical team and perhaps even make them more complacent in performing a meticulous repair”^5^. In this study, HXA-based reconstruction resulted in 0% CSF leakage without the aid of postoperative LD or buttresses for NSF. Permanent and/or temporary mechanical buttresses are recommended, especially in the reconstruction against high-grade CSF leakage^5,14,16,21,34^. The HXA layer acts as a rigid permanent buttress beneath the NSF, similar to harvested bone grafts and synthetic buttresses, while completely sealing the bony defect without CSF spillage. Therefore, the need to compress and stabilize the NSF is reduced, and the role of the NSF is primarily focused on covering the HXA for mucosalization.

This two-institutional study had the inherent limitations of a retrospective study and a small sample size. In addition, there were no cases of large posterior fossa defects in this study population, which is considered the most challenging area^5,16^. Despite such limitations, this study successfully demonstrated that two independent neurosurgeons with limited experience yielded 0% CSF leakage quickly using an HXA-based reconstruction. Although this new technique also requires experience, it significantly shortens the traditional learning curve for skull base reconstruction. However, this study focused only on the outcomes of skull base reconstruction for high-flow CSF leakage. Successful reconstruction is not the only concern in endoscopic skull base surgery, and our results should be carefully interpreted. Nevertheless, based on recent successful results in the literature, including those of the present study, the risk of CSF leakage might be no longer a major hurdle in endoscopic skull base surgery.

## Conclusion

The results of this study showed that the HXA-based skull base reconstruction provides a rigid buttress and minimizes the risk of postoperative CSF leakage. In addition, this technique is reproducible for less-experienced surgeons and can shorten the learning curve. This allows less-experienced surgeons to perform complex procedures with greater confidence and reduced risk, leading to improved patient outcomes and reduced morbidity. 

### Supplementary Information

Supplementary Video 1.

## Data Availability

The datasets analyzed during this study are available from the corresponding author upon reasonable request. Correspondence and requests for materials should be addressed to K.H.K.
